# Wearable skin-like optoelectronic systems with suppression of motion artifacts for cuff-less continuous blood pressure monitor

**DOI:** 10.1093/nsr/nwaa022

**Published:** 2020-02-14

**Authors:** Haicheng Li, Yinji Ma, Ziwei Liang, Zhouheng Wang, Yu Cao, Yuan Xu, Hua Zhou, Bingwei Lu, Ying Chen, Zhiyuan Han, Shisheng Cai, Xue Feng

**Affiliations:** 1 Key Laboratory of Applied Mechanics, Department of Engineering Mechanics, Tsinghua University, Beijing 100084, China; 2 Center for Flexible Electronics Technology, Tsinghua University, Beijing 100084, China; 3 Intensive Care Unit, Beijing Tsinghua Changgung Hospital, Beijing 102218, China; 4 Institute of Flexible Electronics Technology of Tsinghua University, Jiaxing 314000, China

**Keywords:** skin-like devices, optoelectronics, blood pressure monitor, optical measurement

## Abstract

According to the statistics of the World Health Organization, an estimated 17.9 million people die from cardiovascular diseases each year, representing 31% of all global deaths. Continuous non-invasive arterial pressure (CNAP) is essential for the management of cardiovascular diseases. However, it is difficult to achieve long-term CNAP monitoring with the daily use of current devices due to irritation of the skin as well as the lack of motion artifacts suppression. Here, we report a high-performance skin-like optoelectronic system integrated with ultra-thin flexible circuits to monitor CNAP. We introduce a theoretical model via the virtual work principle for predicting the precise blood pressure and suppressing motion artifacts, and propose optical difference in the frequency domain for stable optical measurements in terms of skin-like devices. We compare the results with the blood pressure acquired by invasive (intra-arterial) blood pressure monitoring for >1500 min in total on 44 subjects in an intensive care unit. The maximum absolute errors of diastolic and systolic blood pressure were ±7/±10 mm Hg, respectively, in immobilized, and ±10/±14 mm Hg, respectively, in walking scenarios. These strategies provide advanced blood pressure monitoring techniques, which would directly address an unmet clinical need or daily use for a highly vulnerable population.

## Teaser

We demonstrate skin-like optoelectronic systems that can monitor continuous non-invasive arterial pressure, which would directly address a clinical need or daily use for the population.

## INTRODUCTION

The most reliable method at present for monitoring continuous blood pressure is to insert a catheter sensor into a human artery, which results in massive suffering to the patient [1]. An alternative solution is continuous non-invasive arterial pressure (CNAP) measurement [2–4]. In the 1970s CNAP measurement was already being explored. Until now, some CNAP technologies [5–7] have been proposed and explored [8–13], such as pulse wave velocity [14–18]. These technologies have been exhaustively investigated and optimized over the past 50 years for CNAP measurement. However, there are still no suitable devices for long-term and precise CNAP monitoring in daily use due to the irritation to skin and suppression of motion artifact [19].

Devices for CNAP monitoring will be mounted on human skin for a long time, which requires them to not cause discomfort to the skin [20–23]. Some commercial devices are released for measuring CNAP. These devices need to be bundled or pinched to both fingers and arms [24,25], which results in irritation to human skin. Skin-like devices [26–35], which can be firmly mounted on and deformed with the skin, offer an alternative solution for long-term vital signs in daily use [36–38]. There are some skin-like devices for detecting blood oxygen saturation and pulse rate [39,40]. However, existing strategies for skin-like sensors cannot apply for CNAP monitoring. The certain relationship between CNAP and the pulse transit time (PTT) recording can be set up by the Moens–Korteweg (M–K) equation. PTT refers to the time it takes a pulse wave to travel between two arterial sites [41]. Photoplethysmogram (PPG) detected by optical methods can be used for PTT measurement [42,43], while only one parameter PTT cannot give both systolic and diastolic blood pressure [11,44]. Previous works introduce physical models of the blood circulation system to solve this problem [42,45]. However, these physical models did not consider the influence of the device deformation and motion artifacts. The PPG signals will be mixed with noises caused by the deformation of the device or tissue. It is necessary to build a new physical model for skin-like devices to measure precise PTT.

On the other hand, the PTT is very challenging to measure, as even the ‘onset’ of each pulse is hard to detect without external counter-pressure methods. Generally, the propagating speed of pulse wave velocity (PWV) is over 10 m/s, and the ‘onset’ of each period is difficult to detect due to the influence of motion artifacts [46,47]. The PPG or other pulse-related signals are not subject to the motion artifact or optical interference for measuring accurate PTT. Most artifacts originate in the movement of the distal arterial waveform detector in relation to the skin. There are some designs or algorithms for suppressing the motion artifacts (i.e. template matching, Masimo SET) [48–50]. However, all of them aim at solving motion artifacts for traditional devices. The motion artifacts of skin-like devices are quite different due to the deformable devices. The technologies for detecting distal arterial waveforms such as PPG are based on optical principles, and their accuracy relies on the stability of the optical path [51]. The deformation of skin-like devices will have huge impacts on the optical path. Meanwhile, the deformation of the skin can also change the optical path in the human tissue. False readings caused by shivering or other movements will be more serious in terms of skin-like optoelectronics.

In this work, we propose strategies for skin-like optoelectronic systems that can monitor precise CNAP in daily use. We introduce a theoretical analysis by the virtual work principle for considering the deformation of the skin-like system. The optical difference in the frequency domain is proposed for suppressing the motion artifacts. These features enable the skin-like systems to be mounted on the wrist without irritation and discomfort, and the long-term monitoring of CNAP using wireless data transmission. The assessment of the skin-like systems was made during comparison experiments with invasive (intra-arterial) blood pressure (IBP) monitoring in the intensive care unit (ICU) for >1500 min in total on 44 subjects. The results show that the absolute errors of diastolic and systolic blood pressure were only ±7/±10 mm Hg, respectively, over all subjects. Moreover, the absolute errors slightly increased to ±14 mm Hg in both diastolic and systolic blood pressure during walking.

## RESULTS

### Skin-like systems for precise CNAP monitoring

**Figure 1.**
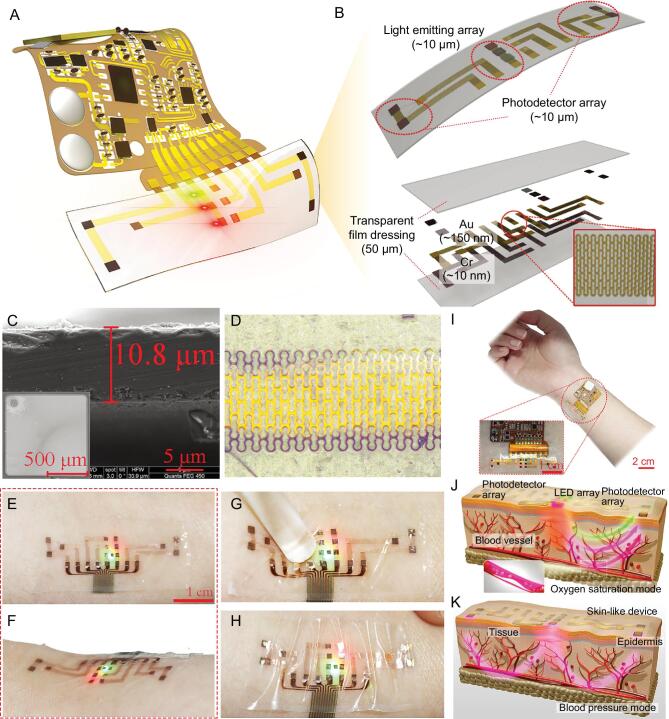
Schematics, designs, optical images and measuring principle of the skin-like wearable systems. (A) Schematic illustration of the skin-like device integrated with a flexible circuit for monitoring CNAP. (B) The front and exploded-view illustrations of the ultra-thin integrated optoelectronic sensor with light-emitting elements with different colors and photodetectors. Inset: the features of the watch-chain interconnect. (C) Scanning electron microscope images showing the surface morphology and thickness of optoelectronic elements. (D) Optical image of the watch-chain interconnect. (E–H) Optical images of the skin-like device mounted on skin, then deformed by being pressed and squeezed. (I) Optical image of the skin-like systems with the flexible circuit of wireless transmission. (J, K) Measuring principle of the skin-like device. Above: optical difference for detecting precise waveform of PPG signals. Below: PTT signal detection by two sets of photodetectors.

The optoelectronic device mainly consists of ultra-thin optoelectronics, watch-chain interconnects and biocompatible package. It is easy to integrate with a flexible circuit to compose a wearable skin-like system, as shown in Fig. 1A and Supplementary Fig. S1. Three inorganic light-emitting elements are in the center of the device, and four photodetectors are placed 1 cm away from both sides of the light-emitting elements, which creates a dual-channel structure for detecting the PPG signals at different location of the body (Fig. 1B). Due to enough distance between light sources and photodetectors, the optical shunt is avoided, which means the light reaches the photodetectors without passing through an arterial bed. The ultra-thin optical semiconductors hybrid-integrated here include infrared light (850 nm, GaAs based), red light (620 nm, GaAs based), green light (515 nm, Al_2_O_3_ based) and photodetector (400–1100 nm, silicon based). All of them are thinned by nano-diamond grinding technology, and the typical thickness is 11 μm/11.7 μm/11.3 μm/11.8 μm, respectively (Fig. 1C and Supplementary Fig. S2). The metal (Cu/Cr) with 160 nm thickness is patterned as a watch-chain shape to serve as electrical interconnects for power and signal transmission, as shown in Fig. 1D. This watch-chain shape has the advantages of low resistance and prominent stretchability. The function layer is packaged into a 50 μm-thick biocompatible material. Here, we utilize the transparent film dressing (W1624, Tegaderm, 3M, USA) as the package, which is widely used to cover and protect wounds and catheters. The device is imperceptible to the skin for long-term use. The thin, soft construction allows the device to make contact with skin only based on van der Waals interactions alone, as shown in Fig. 1E and F. Due to the ultra-thin structure and low modulus mechanics, the device can avoid any significant constraint on natural motions of the skin when undergoing pressing or stretching (Fig. 1G and H).

The skin-like device is easy to connect to a flexible circuit to realize signal processing and wireless transmission. Figure 1I and Supplementary Movie S1 show an example of the flexible system that is able to wirelessly transmit the signals to terminal devices such as a smart phone. Figure 1J illustrates the principle of the light-emitting and receiving sequence to realize the optical difference method. Three light-emitting elements with different wavelengths are derived by pulse light sequentially, which realize the optical difference method against deformation disturbance. The photodetectors can measure the waveforms of the PPG signals at the different locations of the artery (Fig. 1K), and then we can calculate the precise PTT value combined with the optical difference method.

**Figure 2.**
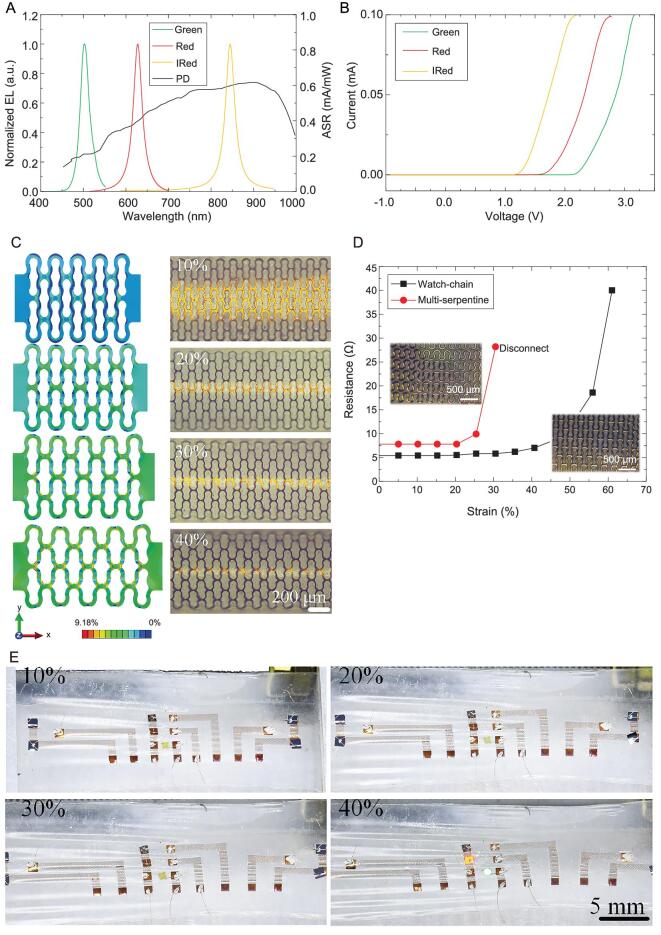
Optical, electrical and mechanical designs of the skin-like wearable device. (A) EL spectra of light-emitting elements (infrared light, red light and green light) and the spectral responsibility of photodetector used in the skin-like device. (B) Current–voltage curves of the ultra-thin light-emitting elements. (C) FEA at the system level and optical images reveal the displacement and strain distributions for uniaxial strains up to 40%. (D) Resistance comparison between six parallel serpentine lines and watch-chain structure. The total width and length of these two structures are the same. Inset: optical images of the two structures. (E) Images of the skin-like device with watch-chain interconnect under different strains after being cycled >300 times.

Light-emitting elements and photodetectors are the inorganic semiconductors based on different materials. When their thickness reaches the level of 10 μm, the semiconductors will become very brittle and hard to be integrated. Here, we use the nano-diamond grinding process to fabricate the ultra-thin optoelectronics with high performance (Supplementary Fig. S3). The fabricating steps start with growing a AlGaInP/AlGaAs, InGaN/GaN quantum well structure epitaxial on a GaAs/sapphire substrate and doping impurity based on the silicon substrate, respectively. Nano-diamond with different sizes (diameter within ±50 nm absolute error) is sorted by centrifugation (Supplementary Table S1) and mixes to a turbid liquid with deionized water (mass ratio of 1:30). Then the optoelectronics are gradually thinned by the turbid liquid. To confirm the performance of thinned optoelectronics, we test the electroluminescence (EL) spectra of light-emitting elements and spectral responsibility of the photodetector, as shown in Fig. 2A and B. The threshold voltages of the light-emitting elements are 2.5, 1.8 and 1.3 V, respectively, and the spectral responsibility arrangement of the photodetector is 400–1200 nm. The critical parameters of the ultra-thin optoelectronics are the same as before the thinning process.

Generally, the shape of the interconnects determines the stretchability of the skin-like device. The most successful shape is the serpentine or serpentine-based pattern. However, the serpentine shape makes the actual length longer, which results in power consumption and signal attenuation. The interconnects for blood pressure monitoring transmit high-frequency signals, and the parasitic capacitance and large resistance will inevitably occur if we still use the serpentine structure. Here, we propose the watch-chain structure to guarantee both stretchability and low impedance. Finite element analysis (FEA) was utilized to analyze the strain distribution during deformation, as shown in Fig. 2C and Supplementary Fig. S4. Full 3D FEA is adopted to analyze the deformation behaviors of the ‘watch-chain’ interconnects under uniaxial stretching. The Cu electrode (thickness 150 nm) is supported by a thin layer of polyimide (PI, thickness 4 μm) and packaged by the film used in our device. The elastic modulus (*E*) and Poisson's ratio (*ν*) of Cu, PI and polyurethane film are 119 GPa/0.34, 2.5 GPa/0.34 and 4.68 MPa/0.5, respectively. Four-node shell and eight-node solid elements are used to analyze the interconnect wires and polyurethane film, and the refined meshes are adopted to ensure accuracy. In the condition of maximum pre-loaded strain up to ∼40%, the maximum strain in the interconnects is limited within 10%, which is also the fracture criteria of Cu [52]. The above analysis demonstrates that the watch-chain structure greatly improves the electronic and mechanical properties of the device. In addition, real-time tests show that the impedance of the watch-chain structure can maintain its impedance unchanged when subjected to a 40% strain. We compare the stretching results of the watch-chain and six parallel serpentine interconnects (Supplementary Fig. S5), as shown in Fig. 2D. Both structures are 1 cm in length and 150 nm in thickness. The total width of six parallel serpentines is the same as the watch-chain shape. The impedance of the watch-chain interconnect is 5.2 Ω, which is 27% smaller than 7.6 Ω of the six serpentine lines. Also, the maximum strain loaded on the watch-chain interconnect can reach 40%, which is larger than the maximum strain of the serpentine line. We repeat loading the applied strain of 0%–40%, and the skin-like device can be cycled for >300 times under this condition without any cracks (Fig. 2E).

### Optical difference in the frequency domain

**Figure 3.**
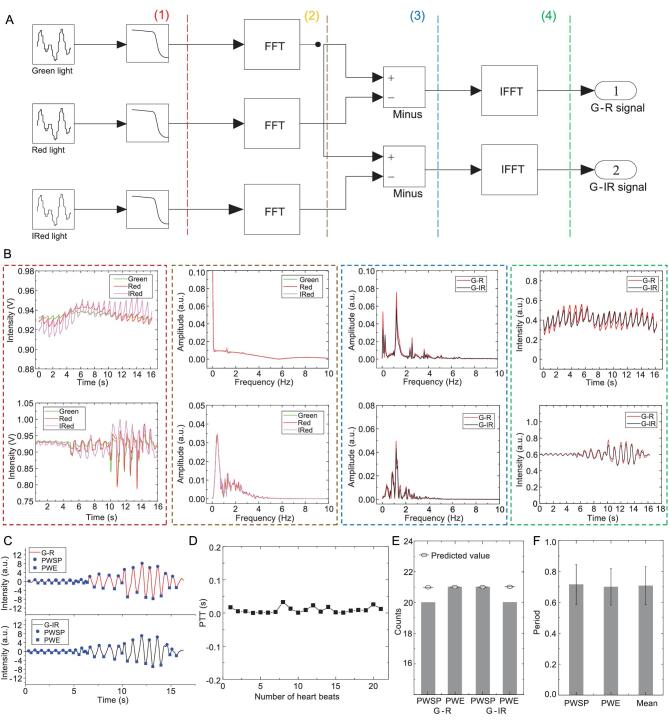
Principle of the optical difference in the frequency domain. (A) Processing steps of the optical difference in the time domain (FFT, fast Fourier transform; IFFT, inverse fast Fourier transform). (B) Two examples of the waveform corresponding to the main steps in optical difference processing, including the stable signals (top) and the signals affected by motion artifact (bottom). (C) PWSP and PWE on G–R (top) and G–IR (bottom) signals obtained by optical difference and bandpass filter. (D) PTT calculated by the systolic peaks in (B). (E) Bar graph of the quantities of peak and valley counts in (B). (F) Period time calculated by PWSE, PWE and their average. Error bars: standard deviation, 20 sets.

Most commonly, problems in PTT measurements arise from motion artifact. False readings of the PTT occur due to the irregular interference caused by movement. This problem is particularly prominent on skin-like devices due to the deformable character. Here, we propose an optical difference in the frequency domain to solve this problem, as shown in Fig. 3A. We list two examples of PPG signals and show the corresponding results processed by the optical difference method, including under the stable and motion artifact situation, respectively (Fig. 3B). The three light-emitting elements are sequentially illuminated for 100 μs at a 2 ms period (Supplementary Fig. S6), and PPG signals corresponding to different light elements are simultaneously detected by the time-multiplex method. The low-pass frequency filter is responsible for suppressing high-frequency noise in circuits, and its filter curve is shown in Supplementary Fig. S7. Generally, the original PPG signals are composed of alternating component (AC) and direct component (DC). AC is caused by the variable, pulsatile component of the light absorption by arterial blood, while DC is made up of the light absorption by tissue, venous blood and non-pulsatile arterial blood. Because the optical path of the light-emitting elements is similar, the significance of the motion artifact is also similar to each light. The AC intensities of each light are quite different when we set the DC at the same level by adjusting the intensity of incident light. Thus, the difference of the PPG intensities between green and red/infrared can keep the useful AC signals as well as effectively suppress the noise caused by motion. The frequency spectrum of each PPG signal is obtained via a fast Fourier transform, and then the amplitude difference between the green and red/infrared spectrum is calculated. At this point, the peaks on the spectrum corresponding to the pulse rate are clearly observed, which approximately equal to 1.35 Hz. Finally, the G–R and G–IR signals are converted from the frequency domain to the time domain by inverse fast Fourier transform. A homemade algorithm can achieve the optical difference method, and the flow chart is shown in Supplementary Fig. S8. We also use the sine wave signals with white noise to demonstrate efficiency. As shown in Supplementary Fig. S9, the original signals are unable to read the 1 Hz frequency even with a band-pass filter, while the optical difference in frequency can recover the periodic signal. Thus, the PPG signals are successfully separated from the motion artifact, as shown in Fig. 3C. Then we can easily obtain the precise time points of the pulse wave systolic peak (PWSP) and pulse wave end (PWE), which can be used to calculate the PTT value (Fig. 3D). The peak and valley count of the two signals are the same as the predicted values, as shown in Fig. 3E. The average periodic time of peak–peak, valley–valley and pulse are near 0.67 s as shown in Fig. 3F, which corresponds to the frequency of the pulse rate.

### Assessments in ICU

We propose a new model using virtual work to connect PTT and CNAP. Blood vessels are subjected to three types of stresses that are independent of the radius of the vessels. They are the external strains by other tissues (e.g. muscles), blood pressure and the stresses resulting from the strain of blood vessels themselves. The relationship between these three stresses near PWSP and PWE can be expressed by the virtual work equation:

(1) \begin{equation*}{P_{b\!p}} - \frac{{2\rho }}{{P\!T\!{T^2}}}\left(1 - \frac{{{r_0}}}{r}\right) - {P_{e\!x}} = 0.\end{equation*}

In equation (1), *P_bp_* is the instant blood pressure to the blood vessel; *ρ*, *r*_0_ and *r* are the blood density, initial and current vessel radius, respectively; *P_ex_* is the external pressure to the vessel by other tissues, including the pressure caused by motion artifact. Then we can calculate the blood pressure via equation (1) and the Beer–Lambert law:

(2) \begin{eqnarray*} &&{P_{S\!B\!P}} &=& {A_1} + {B_1}\!\ln ({I_{DC}}\!) \nonumber\\ &&+\, \frac{{{C_1}}}{{P\!T\!{T^2}}}\left[1 - \frac{{{D_1}}}{{C\!\ln ({{{I_{\mathit {min} }}} /{I_{DC}}}\!)}}\right], \end{eqnarray*}

(3) \begin{eqnarray*} &&{P_\mathit{DBP}} &=& {A_2} + {B_2}\!\ln ({I_{DC}}) \nonumber\\ &&+\, \frac{{{C_2}}}{{P\!T\!{T^2}}}\left[1 - \frac{{{D_2}}}{{C\!\ln ({{{I_{\mathit {max} }}} /{I_{DC}}}\!)}}\right], \end{eqnarray*}

where *A*_1,2_, *B*_1,2_, *C*_1,2_ and *D*_1,2_ are constants that can be calibrated by experiments, and the detailed theoretical derivation can be seen in the Methods section.

The skin-like system assessments are made on-site in Beijing Tsinghua Changgung hospital. The ICU is prospectively selected to record IBP. All the patients give written informed consent, which is approved by the ethics committee of the Tsinghua Changgung Hospital (Approval No. 18083-0-02). We use a closed-loop blood sampling system (682051, Argon, USA) and patient monitor (MX600, Philips) to record the IBP (Supplementary Fig. S10). This blood sampling system is certificated by the Gabarith test. The blood pressure is already monitored by an arterial cannula in the vessel. Drugs that may have a potential risk of changing the elasticity of blood vessels are not used during the experiments. Subjects keep the supine position, and both upper arm or wrist are at heart level. Thus, no potential energy affects the value of blood pressure. The information for each patient, including age, sex and breathing status, is recorded from their medical identifications, as shown in Supplementary Table S2. The skin-like system is mounted near the position of the radial artery, and IBP is recorded by the patient monitor for verifying the accuracy of the skin-like systems (Supplementary Fig. S11). The dual PPG signals monitored by our skin-like systems are synchronized with IBP by a time marker.

We recorded the IBP and CNAP monitored by our skin-like system on 44 subjects over 1500 min. The estimated systolic and diastolic blood pressures are summarized in Table 1 and Supplementary Table S3, respectively. In general, the absolute errors of the diastolic blood pressure (DBP) are smaller than those of systolic blood pressure (SBP), because the value of DBP is smaller than SBP. The correlation coefficients of SBP between estimated and invasive values range from 0.62 to 0.96, and their mean value and standard deviation (SD) are 0.82 and 0.08, respectively. The root mean square of estimation errors (RME) values of the 44 patients vary from 0.04% to 7.08%, and the mean value is 3.05%. The probability distribution of errors is also evaluated. Out of 50 000 heartbeats from 44 patients, 94% of the estimates were within the error range of 5 mm Hg, 4% were within 5–10 mm Hg, while only 1% of the absolute error was larger than 10 mm Hg.

**Table 1.** Statistical results of CNAP monitor from 44 subjects, where the estimated systolic blood pressure values are compared with IBP, each subject being tested for >30 min.

	Estimation error (%)			Error distribution (%)^d^		
Subject No.	CC^a^	RME^b^	Mean^c^	<5%	<10%	≥10%

1	0.88	4.35	2.38	84.6	14.4	1
2	0.84	2.35	0.02	98	2	0
3	0.81	2.15	–0.29	99	1	0
4	0.92	6.92	2.61	56.4	36.4	7.2
5	0.8	3.8	0.83	96.1	3.9	0
6	0.87	2.49	–0.14	97.8	2.2	0
7	0.87	3.23	–1.5	98	2	0
8	0.76	1.67	–0.87	100	0	0
9	0.77	2.95	–1.04	93.9	6.1	0
10	0.69	2.73	–0.31	97.6	2.4	0
11	0.75	3.9	–0.23	81.3	18	0.7
12	0.88	2.84	–1.27	95.8	4.2	0
13	0.64	2.9	1.42	99.8	0.2	0
14	0.89	1.87	0.7	100	0	0
15	0.83	1.04	–0.35	97.5	2.5	0
16	0.86	2.41	–0.51	100	0	0
17	0.86	2.93	1.95	99.4	0.6	0
18	0.75	3.12	1.24	99.5	0.5	0
19	0.96	3.99	1.15	98.5	1.5	0
20	0.94	4.03	–1.25	96.3	3.7	0
21	0.73	2.53	0.32	100	0	0
22	0.72	3.07	–0.19	100	0	0
23	0.74	2.55	–1.21	100	0	0
24	0.89	1.83	0.28	100	0	0
25	0.85	1.83	–0.07	98.5	1.1	0.4
26	0.73	1.71	1.03	99.4	0.6	0
27	0.75	2.54	1.24	95.6	4.1	0.3
28	0.78	1.75	0.26	100	0	0
29	0.73	6.7	0.42	75.7	20.4	3.8
30	0.9	2.77	0.47	99.9	0.1	0
31	0.69	5.37	1.61	74.4	22.6	3
32	0.93	7.08	–3.1	61.9	30.2	8
33	0.8	4.54	1.61	67.6	24.6	7.7
34	0.91	2.43	0.22	97.6	2.4	0
35	0.62	4.15	2.43	83.2	15.1	1.7
36	0.93	1.94	1.03	99.9	0.1	0
37	0.85	2.06	0.51	98.9	1.1	0
38	0.78	2.36	–0.67	100	0	0
39	0.93	4.19	1.29	93.6	6.4	0
40	0.77	2.54	–0.57	99.6	0.4	0
41	0.8	3.46	–1.53	94.1	5.9	0
42	0.8	3.35	0.71	95.8	4.2	0
43	0.88	1.67	–0.07	97.6	2.4	0
44	0.84	0.23	0	100	0	0
Max^e^	0.96	7.08	2.61	100.00	36.40	8.00
Min^f^	0.62	0.23	–3.10	56.40	0.00	0.00
Avg^g^	0.82	3.05	0.24	93.70	5.53	0.77
SD^h^	0.08	1.45	1.18	10.80	8.92	2.03

^a^CC, correlation coefficient between estimated and invasive values. ^b^RME, root mean square of estimation errors. ^c^Mean, arithmetic means of estimation errors. ^d^Error distribution <5%, <10%, ≥10% represents range of normalized error within 5%, 10% and greater than 10%. ^e^Max, maximum value. ^f^Min, minimum value. ^g^Avg, average value. ^h^SD, standard deviation among 44 patients.

**Figure 4.**
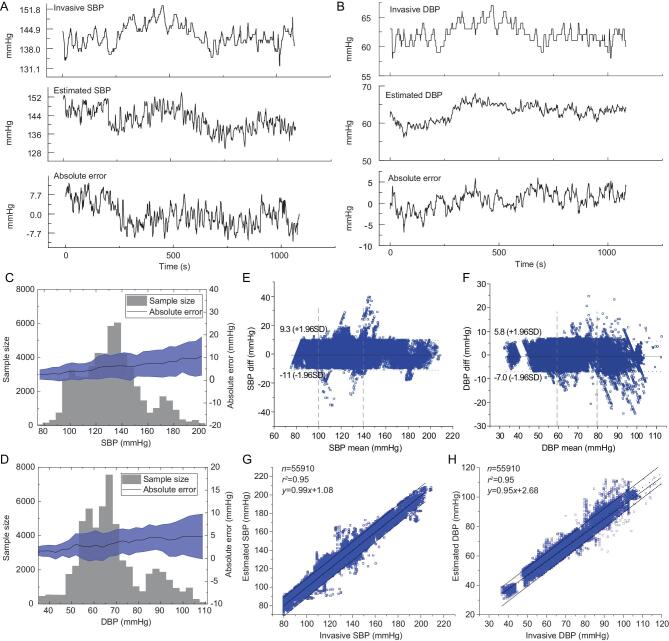
Temporal course of (A) SBP and (B) DBP monitored by blood sampling system, skin-like systems, and their absolute errors from one subject. (C, D) Sample size, error distribution and error standard deviation of each section of SBP and DBP. (E, F) Plots of the pressure difference between IBP and CNAP monitored by the skin-like systems, and the mean pressure for IBP and CNAP, in 44 participants for SBP and DBP (the number of data points is 55 910 for each). The two vertical lines divide the pressure scale into low, medium and high regions as defined in the clinic. (G, H) Plots of IBP versus CNAP monitored by skin-like systems. The dotted line and center straight line represent theoretical and fitting linear regression, respectively.

Figure 4A and B, and Supplementary Fig. S12 illustrate the CNAP monitored by the skin-like system and the IBP, respectively, as well as the estimation errors from a representative subject. The errors of SBP and DBP do not exceed 10 mm Hg in the comparison test over 1000 s. There is no significant difference between the CNAP and IBP. Figure 4C and D shows the CNAP distribution, the corresponding absolute errors, and the standard deviation (SD) of the error. The sampled SBP and DBP values range widely from 80/40 mm Hg to 200/110 mm Hg, respectively. The measuring range of the experiment covers the scale from low to ultra-high blood pressure as defined in the clinic. The absolute error and standard deviation are positively correlated with the blood pressure value, and the maximum average error is ∼10 mm Hg. Figure 4E and F shows the Bland–Altman plots corresponding to the monitored results, respectively. Here, the difference of over 55 000 values is plotted against the average values (in millimeters of mercury). Reference lines are marked to show ±1.96 SD. The ±1.96 SD is 11/7 mm Hg respectively, which means the construction of approximate 95% confidence intervals. In our tests, the overall correlation coefficient is 0.95, and the typical error is <10 mm Hg and <5 mm Hg, respectively.

The absolute errors of both SBP and DBP are less than ±10 mm Hg, and the data from skin-like systems follow a similar trend as those measured by the blood sampling system. Overall, only 2.05% points of systolic pressure (SP) and diastolic pressure (DP) are outside the 95% limits of agreement. The average difference between IBP and CNAP is <1 mm Hg, as shown in Fig. 4G and H. These results demonstrate the agreements of the two devices are also well.

**Figure 5.**
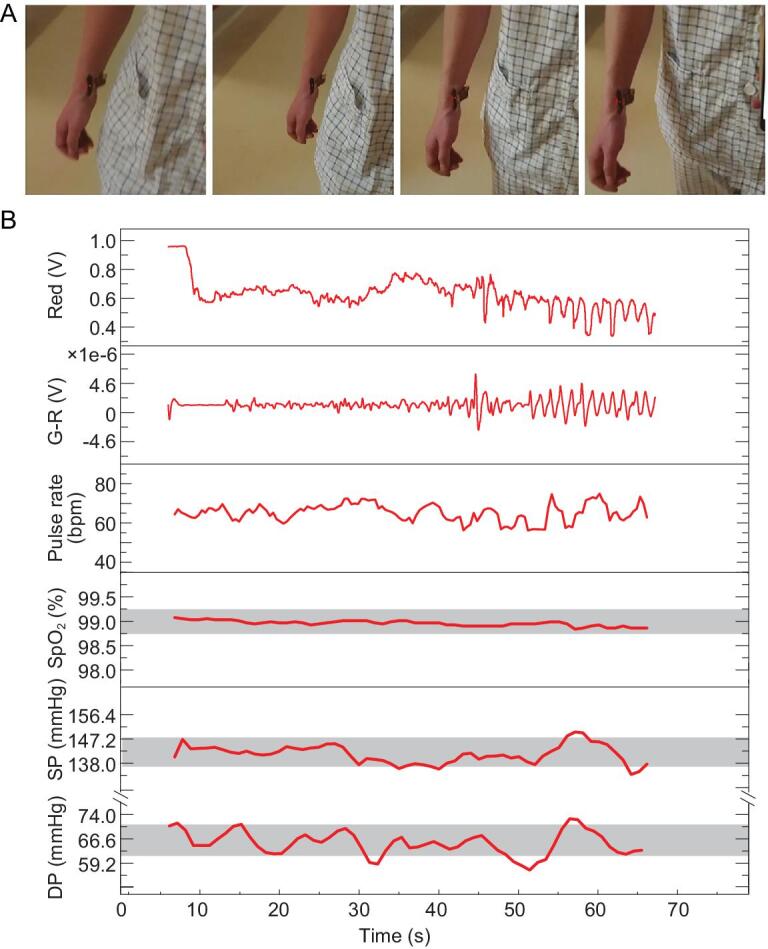
Skin-like systems mounted on the wrist to detect the subject's physiological information while walking. (A) Images of the subject's wrist to which were attached the skin-like systems during walking. The skin-like systems were smeared by optical filter dye to limit the ambient light interference. (B) The signal of original PPG, optical difference, pulse rate, SpO_2_ and blood pressure monitoring during subject walking. Grey blocks: ±0.5% and ±10 mm Hg error.

Moreover, vital signs, including pulse rate, blood oxygen saturation (SpO_2_) and blood pressure, were monitored simultaneously in the walking scenario, as shown in Fig. 5. Also, we tested the performance with sitting and standing, as shown in Supplementary Fig. S13. In the experiments, one subject with the skin-like wearable systems mounted on the wrist was instructed to walk with arm movement. During walking, the PPG signals were recorded and wirelessly transmitted to a host terminal (Fig. 5A, Supplementary Movie S2). The original PPG signal, G–R signal, pulse rate, SpO_2_ and blood pressure are shown in Fig. 5B. Because the traditional devices, including fingertip oximeter and sphygmomanometer, cannot measure vital signs precisely during human walking, we monitored the SpO_2_ and blood pressure before walking as the standard value. During walking, the period of pulse is clearly shown in the G–R signal, which is 70 bpm. The SpO_2_ values by skin-like systems are near 98.8% during walking, and the maximum absolute error is less than ±1%. The monitored SP and DP fluctuate near 143 mm Hg and 66 mm Hg, respectively, and the maximum absolute error is less than ±14 mm Hg. For the blood pressure monitor, these are acceptable errors because the arm swing during walking yields a strong noise signal. The results demonstrate that the system can measure vital signs precisely and with no irritation to human activities.

## DISCUSSION

CNAP has found its greatest utility as an early warning of cardiovascular disease. Our skin-like systems are an excellent tool. Here we propose skin-like optoelectronics systems for precise CNAP monitoring. The stable optical measurements for the skin-like optoelectronics system are investigated. We use the virtual work principle to analyze the stress on the blood vessel, and build up the relationship of blood pressure, PTT and PPG. This theoretical analysis is the foundation of precise CNAP monitoring. The optical difference in the frequency domain is an efficient solution for improving the performance of skin-like optoelectronics. The skin-like systems can be used for long-term monitoring in the emergency or daily settings without any irritation to the skin. Risks of allergy or other adverse reactions are minimized due to the use of compatible materials for the package. The skin-like systems are demonstrated by monitoring the blood pressure of 44 subjects over 1500 min, and the errors are acceptable in terms of clinical diagnosis.

## CONCLUSION

The skin-like systems are highly versatile for applications such as monitoring ICU patients or evaluating physical conditions in daily life. Combined with a wireless many-to-one receiving terminal, the systems are convenient to report all patients’ situations to the doctor simultaneously. Thereby, the systems can serve as an early-warning functionality in terms of cardiovascular disease or potential patients. For example, the skin-like systems can be used as a precautionary monitor to prevent chronic or sudden diseases such as cardiac arrest. The same platforms can also be applied to an exercise system or sports competition to feedback every athletic physiologic parameter and guide earlier supplementation. In these scenarios or other interests, the monitoring data could be accumulated and provide information for big data analysis. The skin-like systems could be broadly utilized and change the quality of our life in different aspects, and open up a new prospect in biomedical engineering for both hospital diagnostic and daily healthcare.

## METHODS

**Figure 6.**
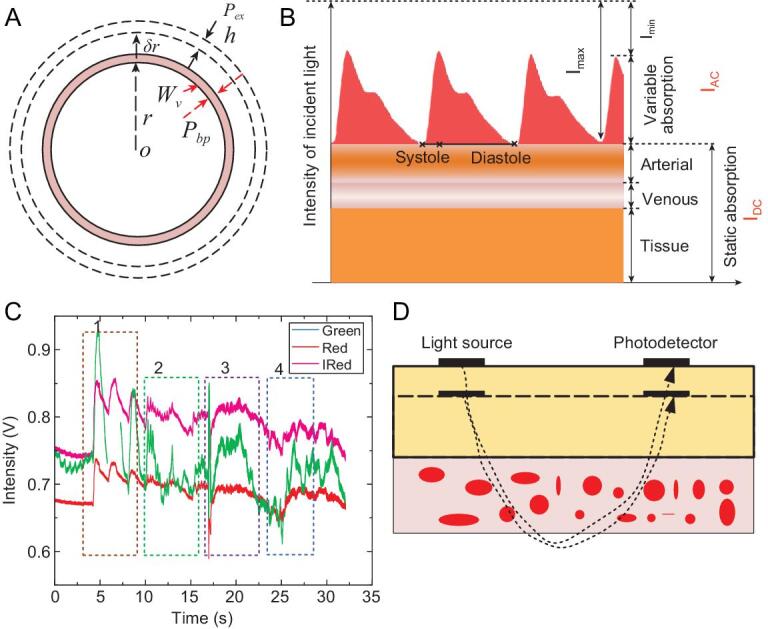
The theoretical model for analyzing the variation of blood pressure by the virtual work principle. (A) The parameters used to analyze the real stress acting through the virtual displacement of the blood radius *δr*. *r* and *h*: radius and thickness of the blood vessel, respectively; *P_bp_* and *P_ex_*: blood pressure to the blood vessel and the external pressure by other tissue, respectively. (B) A model for the light transmission path through pulsating arterial blood, non-pulsating arterial blood, venous blood and other tissues over several cardiac cycles. *I_AC_*: alternating current; *I_DC_*: direct current [30]. Copyright 2014, Wiley. (C) Typical PPG signals with motion artifact, including slow and fast squeezing of the skin-like systems, bending the wrist and clenching fist, corresponding to sections 1–4. (D) Schematic of the optical path and tissue changed by external stress.

In human tissue, the blood vessels are always subjected to three forces no matter how the blood pressure changes, as shown in Fig. 6A. These forces are produced by blood pressure (*P_bp_*), external pressure (*P_ex_*) by tissue, and the stress ($W_v$) in the blood vessel. The blood vessels can be treated as isotropic linear elastic material with a round shape. The blood vessel will be a stable state with a certain volume of internal blood. Under this situation, the virtual work equation can be expressed by:

(M1) \begin{eqnarray*} \int_{s}{{{P_{bp}}\cdot \delta r}}\!\mathit{ds} + \int_{v}{{W_v}\cdot \delta \varepsilon dv} \nonumber\\ \quad +\, \int_{s}{{P_{e{\!}x}}\cdot \delta r\!\mathit{ds} = 0}, \end{eqnarray*}

where *P_bp_* is real-time blood pressure, *δr* is the virtual change of the radius, *s, ϵ* and $v$ are the surface of the blood vessel, strain in the blood vessel, and volume of the blood vessel, respectively.

When the radius *r* changes to *r + δr*, the virtual work made by the three forces can be calculated as follows. The first term related to *P_bp_* is:

(M2) \begin{eqnarray*} \int_{s}{{{P_{b\!p}}\cdot \delta r}}\!\mathit{ds} = \int_{r}^{{r + \delta r}}{{{P_{bp}}\cdot (2\pi\! Lx)}}dx \nonumber\\ \quad = \pi\! {P_{bp}}\!{x^2} | {_r^{r + \delta r}} = \pi\! L\!{P_{bp}} ( {2r\!\delta r + \delta {r^2}}), \nonumber\\ \end{eqnarray*}

where *L* is the length of the blood vessel. The *δr^2^* is a second-order small value, and the equation (M2) can be shortened as follows:

(M3) \begin{equation*}\int_{s}{{{P_{bp}}\cdot \delta r}}\!\mathit{ds}= 2\pi\! L\!{P_{bp}}r\!\delta r.\end{equation*}

The fundamental principle of the PTT-based method is based upon the pulse wave velocity (PWV) recording through the Moens–Korteweg (M–K) equation:

(M4) \begin{equation*}P\!W\!V = \frac{K}{{P\!T\!T}} = \sqrt {\frac{{Eh}}{{\rho d}}}, \end{equation*}

which relates PWV and PTT with the elastic modulus of the vessel wall *E*, blood density *ρ*, and arterial dimension properties, such as vessel thickness *h* and arterial diameter *d*. PWV is inversely related to PTT (PWV = *K*/PTT), where *K* is the distance between the two certain peripheral sites. The second term in equation (M1) can be expressed as follows:

(M5) \begin{eqnarray*} \int_{v}{{{W_v}\cdot \delta \varepsilon dv}} = \int_{r}^{{r + \delta r}}{{E\cdot A\frac{{x - {r_0}}}{{{r_0}}}}}d(2\pi x) \nonumber\\ \quad = \int_{r}^{{r + \delta r}}{{\frac{{2\rho {r_0}}}{{P\!T\!{T^2}h}}\cdot hL\frac{{x - {r_0}}}{{{r_0}}}}}d(2\pi x). \nonumber\\ \end{eqnarray*}

In equation (M5), *A* and *r*_0_ represent the cross-sectional area of the blood vessel. The vessel is a film material and its thickness *h* is similar to constant when the *r* changes. Equation (M5) can be compacted as equation (M6):

(M6) \begin{equation*}\int_{v}{{{W_v}\cdot \delta \varepsilon dv}} = \frac{{4\pi \rho L}}{{P\!T\!{T^2}}}(r - {r_0})\delta r.\end{equation*}

The third term in equation (M1) is similar to the first term in equation (M6):

(M7) \begin{equation*}\int_{s}{{{P_{\mathit {ex}}}\cdot \delta r}}\!\mathit{ds} = 2\pi\! L\!{P_{\mathit{ex}}}r\delta r.\end{equation*}

Now the equation (M1) can be written as equation (M8):

(M8) \begin{eqnarray*} 2\pi\! L\!{P_{bp}}r\!\delta r - \frac{{4\pi \rho L}}{{P\!T\!{T^2}}}(r - {r_0})\delta r \nonumber\\ \quad -\, 2\pi\! L\!{P_{\mathit{ex}}}r\delta r = 0. \end{eqnarray*}

Here we define the direction away from the center of the blood vessel as positive. Equation (M8) can be compacted to equation (M9):

(M9) \begin{equation*}{P_{bp}} - \frac{{2\rho }}{{P\!T\!{T^2}}}\left(1 - \frac{{{r_0}}}{r}\right) - {P_{e\!x}} = 0.\end{equation*}

Each emitting light will be through a cutaneous vascular bed and then reflect to the photodetector during the detection of the PPG signal. The light absorption by blood and other tissue substances determines the pulse-add component of the signal, as shown in Fig. 6B. The fundamental principle of the optical absorption in tissue is based upon the Beer–Lambert law:

(M10) \begin{equation*}{I_{out}} = {I_{\mathit{in}}}{e^{\!{c_0}{\varepsilon _0}{L_0}}}{e^{\!{c_1}{\varepsilon _1}{L_1}}} = {I_{DC}}{e^{{c_1}{\varepsilon _1}{L_1}}},\end{equation*}

where *I_out_*, *I_in_* and *I_DC_* represent the output light intensity, the intensity of the incident light, and the tissue absorbed intensity, respectively; *c*_1_, *ϵ*_1_, and *L*_1_ are the average substance concentration in blood, index of light absorption, and light path in the blood. If the light source and photodetector are not very close, the light will totally cross the blood vessel and then turn back, as shown in Fig. 6D. The light path *L*_1_ will be proportional to the radius of the blood vessel:

(M11) \begin{equation*}r = C\cdot {L_1}.\end{equation*}

*C* represents a certain constant in equation (M11). The radius *r* can be calculated by equation (M10) and (M11).

In a PPG period, the maximum and minimum intensity are corresponding to DBP and SBP. The *P_bp_* can be obtained with equations (M9) and (M12), as shown in equation (M13):

(M12) \begin{equation*}r = \frac{C}{{{c_1}{\varepsilon _1}}}\ln \left(\frac{{{I_{o\!u\!t}}}}{{{I_{D\!C}}}}\right),\end{equation*}

(M13) \begin{eqnarray*} {P_{bp}} = {P_{e\!x}} + \frac{{2\rho }}{{P\!T\!{T^2}}}\left[1 - \frac{{{r_0}{c_{\!1}}{\varepsilon _1}}}{{C\!\ln ({{{I_{out}}} /{I_{DC}}}})}\right]. \nonumber\\ \end{eqnarray*}

The motion artifact will introduce the extra pressure to the external surface of the blood vessel. Figure 6C represents typical PPG signals with motion artifact. *I*_*DC*_ fluctuations are particularly large. That means the main influences of the motion focus on the outside of the blood vessel. Thus, we can add extra pressure in equation (M13) to reflect the motion artifact:

(M14) \begin{eqnarray*} &&{P_{bp}} &=& {P_{ex}} + {C_m}\!\ln ({I_{D\!C}}) + \frac{{2\rho }}{{P\!T\!{T^2}}} \nonumber\\ &&\times\, \left[1 - \frac{{{r_0}{c_{\!1}}{\varepsilon _1}}}{{C\!\ln ({{{I_{out}}} /{{I_{DC}}}})}}\right], \end{eqnarray*}

where the added term is proportional to *I_DC_*, as shown in Fig. 6D.

*C_m_* is constant. Then we can obtain the equations about SBP and DBP:

(M15) \begin{eqnarray*} &&{P_{S\!B\!P}} &=& {A_1} + {B_1}\!\ln ({I_{DC}}\!) + \frac{{{C_1}}}{{P\!T\!{T^2}}}\nonumber\\ &&\times\, \left[1 - \frac{{{D_1}}}{{C\!\ln ({{{I_{\mathit{min} }}} /{{I_{DC}}}}\!)}}\right], \end{eqnarray*}

(M16) \begin{eqnarray*} &&{P_{D\!B\!P}} &=& {A_2} + {B_2}\!\ln ({I_{DC}}\!) + \frac{{{C_2}}}{{P\!T\!{T^2}}}\nonumber\\ &&\times\, \left[1 - \frac{{{D_2}}}{{C\!\ln ({{{I_{\mathit{max} }}} /{{I_{DC}}}}\!)}}\right]. \end{eqnarray*}

*A*_1,2_, *B*_1,2_, *C*_1,2_ and *D*_1,2_ are certain constants that can be calibrated by experiments, and the SBP and DBP can be derived through equations (M15) and (M16).

## Supplementary Material

nwaa022_Supplemental_File
